# Pinafore up to Date*“Leads” should be costumed in black gowns—kimono effect—bordered with deep crimson. Chorus should be costumed in sack-cloth, with fool’s caps and baby rattles.

**Published:** 1914-01

**Authors:** G. Frank Lydston


### Abstracts and Selections.

The Inimitable, Indomitable and Irrepressible Lydston sends us the following clever parody. The significance of the Cockney dialect will be understood when it is known that the President of the Royal College of Surgeons of England visited Chicago, to "tell 'em how," and the A. C. S. makes it a point to pattern after the R. C. S. of England. Lydston says, "There was a negro among the Chicago bunch of 'Fellows.' " (A negro fellow. D.)

### Pinafore up to Date.*

BY G. FRANK LYDSTON, M. D.

Respectfully dedicated to Sir Bombasticus A Byrdd, F. R. A. C. S. S. (Royal American College of Surgical Snobs), Sir C. Putting-it Over-Smoothe, F. R. A. C. S. S., Sir O. Lord D. Liverus, F. R. A. C. S. S., and Sir B. Con-Bunke, F. R. A. C. S. S.

I.

When I was young I served h'a term
H'as a poor little fag of an 'ospital 'terne;
H'analyzed h'urine an' a 'ole lot more;
Did h'everything but sweep th' bloody floor.
I did me job with such faithfulness,
That now I h'am a Fellah of the h'A. C. h'S.

*"Leads" should be costumed in black gowns—kimono effect—bordered with deep crimson. Chorus should be costumed in sack-cloth, with fool's caps and baby rattles.

Chorus.

'E did 'is job with such faithfulness,
That now 'e h'is a Fellah of the h'A. C. h'S.

II.

I nevah got a chawnce to h'operate,
H'an' many a time I cussed me fate,
I 'itched me bloomin' drag to politics,
H'an' bally well showed 'em some bettah tricks,
I put it h'over with such clevahness,
That now I h'am a Fellah of the h'A. C. h'S.

Chorus.

'E put it h'over with such clevahness,
That now 'e h'is a Fellah of the h'A. C. h'S.

III.

H'if the othah fellah gets h'a case,
Look for the royal stamp h'on 'is face,
H'an if 'e 'asn't got me seal h'on 'is mug,
Call for a bobby h'an' put 'im h'in the jug.
Then keep 'im h'in the vile duress,
To make 'im pay h'attention to the h'A. C. h'S.

Chorus.

Then keep 'im h'in the vile duress,
To make 'im pay h'attention to the h'A. C. h'S.

IV.

For many years I split me fee,
But the blawsted layman got h'onto me,
So I'm reformed h'an gettin' very good,
I'll make th' othah fellah be'ave as 'e should
The h'old, h'old game's played out, don'tcher see;
Climb h'aboard the wagon an' get yer degree.

Chorus.

The h'old, h'old game's played out, don'tcher see;
Climb h'aboard the wagon an' get yer degree.

V.

'Ope you don't think I put it h'over raw,
H'incorporatin' nev'vys and sons-h'in-law,
Like h'all us blokes they're famous, don't yer see,
You'll find h'every name h'in the di-rec-toree.
So don't feel 'ard h'an kick h'up a muss,
Just round h'up yer cases h'an' send 'em h'all to us.

Chorus.

So don't feel 'ard h'an kick h'up a muss,
Just round h'up yer cases h'an' send 'em h'all to us.

VI.

Now kind friends h'if you'll listen to me,
I'll tell you h'all 'ow you can get to be,
H'a devil of a fellah h'an' a h'A. C. h'S.
Get in h'on me charter h'an' nevah mind the rest.
The little chaps get nothing,' don'tcher know,
Never give the rummies h'a chawnce for to grow.

Chorus.

The little chaps get nothin', don'tcher know,
Never give the rummies h'a chawnce for to grow.

